# Optimized Adenoviral Vector That Enhances the Assembly of FMDV O1 Virus-Like Particles *in situ* Increases Its Potential as Vaccine for Serotype O Viruses

**DOI:** 10.3389/fmicb.2020.591019

**Published:** 2020-11-04

**Authors:** Micaela Ziraldo, Juan E. Bidart, Cecilia A. Prato, María V. Tribulatti, Patricia Zamorano, Nora Mattion, Alejandra L. D’Antuono

**Affiliations:** ^1^Centro de Virología Animal, Consejo Nacional de Investigaciones Científicas y Técnicas, Buenos Aires, Argentina; ^2^Instituto de Virología e Innovaciones Tecnológicas, Centro de Investigaciones en Ciencias Veterinarias, Instituto Nacional de Tecnología Agropecuaria, Consejo Nacional de Investigaciones Científicas y Técnicas, Buenos Aires, Argentina; ^3^Laboratorio de Inmunología Molecular, Instituto de Investigaciones Biotecnológicas, Universidad Nacional de San Martín, Consejo Nacional de Investigaciones Científicas y Técnicas, Buenos Aires, Argentina

**Keywords:** foot-and-mouth disease virus, replication-defective human adenovirus type 5, genetic vaccine, mutated VP2 capsid protein, virus-like particles O1/Campos, neutralizing antibodies

## Abstract

Although replication-defective human adenovirus type 5 (Ad5) vectors that express *in situ* the capsid-encoding region of foot-and-mouth disease virus (FMDV) have been proven to be effective as vaccines in relevant species for several viral strains, the same result was not consistently achieved for the O1/Campos/Brazil/58 strain. In the present study, an optimization of the Ad5 system was explored and was proven to enhance the expression of FMDV capsid proteins and their association into virus-like particles (VLPs). Particularly, we engineered a novel Ad5 vector (Ad5[P_VP2_]_OP_) which harbors the foreign transcription unit in a leftward orientation relative to the Ad5 genome, and drives the expression of the FMDV sequences from an optimized cytomegalovirus (CMV) enhancer-promoter as well. The Ad5[P_VP2_]_OP_ vaccine candidate also contains the amino acid substitutions S93F/Y98F in the VP2 protein coding sequence, predicted to stabilize FMD virus particles. Cells infected with the optimized vector showed an ∼14-fold increase in protein expression as compared to cells infected with an unmodified Ad5 vector tested in previous works. Furthermore, amino acid substitutions in VP2 protein allowed the assembly of FMDV O1/Campos/Brazil/58 VLPs. Evaluation of several serological parameters in inoculated mice with the optimized Ad5[P_VP2_]_OP_ candidate revealed an enhanced vaccine performance, characterized by significant higher titers of neutralizing antibodies, as compared to our previous unmodified Ad5 vector. Moreover, 94% of the mice vaccinated with the Ad5[P_VP2_]_OP_ candidate were protected from homologous challenge. These results indicate that both the optimized protein expression and the stabilization of the *in situ* generated VLPs improved the performance of Ad5-vectored vaccines against the FMDV O1/Campos/Brazil/58 strain and open optimistic expectations to be tested in target animals.

## Introduction

Included in the A list of infectious diseases of animals of the Office International des Épizooties (OIE), foot-and-mouth disease (FMD) is one of the most contagious animal illnesses that affects several species of wild and domestic cloven-hoofed ruminants (Knight-Jones et al., 2016, 2017). Although the disease has been circulating for a long time, it remains of major concern to livestock farmers and official health authorities since it causes a huge socioeconomic impact, particularly in the agricultural sector. Due to the financial losses incurred, FMD is still considered the major disease concerning production and international trade in foodstuffs of animal origin, food safety, and economic development, affecting both small- and large-scale production (Knight-Jones and Rushton, 2013).

The causative agent, FMD virus (FMDV), is a positive-sense single-stranded RNA virus that belongs to the genus *Aphthovirus* of the Picornaviridae family. The World Reference Laboratory for FMD lists seven FMDV serotypes [Euroasiatic serotypes O, A, Asia1, and C and South African territories (SAT) serotypes SAT1, SAT2, and SAT3] in seven regional endemic pools, among which no cross-serotype protection is expected (FAO, 2020). Within the seven serotypes described, serotype O is the most prevalent and is distributed across six out of the seven described endemic pools (Brito et al., 2017). Furthermore, serotype O was responsible for the recent FMD outbreaks in Colombia (2017–2018), Ecuador (2011), and Venezuela (2013) (Centro Panamericano de Fiebre Aftosa, OPS, and OMS., 2019).

In FMD endemic regions, disease control is mainly achieved by regular vaccination (Knight-Jones and Rushton, 2013; Naranjo and Cosivi, 2013). In this regard, the currently employed vaccines are reliant on the use of highly purified, highly concentrated, inactivated FMDV antigen (Grubman and Baxt, 2004). Despite their effectiveness, these vaccine formulations have several drawbacks, including the difficulty to differentiate infected from vaccinated animals, the need for expensive high-containment Biosafety Level 4-OIE (BSL4-OIE) manufacturing facilities, and the generation of large amounts of infectious virus required for vaccine production. Another problem associated with the traditional FMD vaccine is the risk of live virus release during the manufacturing process or the unintentional use of incompletely inactivated products. These disadvantages highlight the need for new-generation genetically engineered vaccines.

At the moment, the most successful strategy in the development of new FMDV vaccines has been the production of a replication-defective human serotype 5 adenovirus (Ad5) that delivers the capsid coding region of FMDV along with the viral protease 3C (3C^pro^ ), required for its processing (P12A3C cassette). Initial studies reported by Mayr et al. (1999) have shown that an Ad5 vector that delivers the capsid and 3C^pro^ coding regions of FMDV A12/119/Kent/UK/32 (Ad5-A12) was effective at protecting swine from clinical disease after direct contact challenge. Subsequently, it was demonstrated that one dose of Ad5-A24, which contains the P12A3C cassette from FMDV A24/Cruzeiro/BRA/55, confers early protection against homologous FMDV challenge in swine (Moraes et al., 2002) and cattle (Pacheco et al., 2005; Schutta et al., 2016). More recently, it has been shown that an Ad5 expressing FMDV O/Manisa/TUR/69 antigen (Ad5-O1Man) could fully protect swine at 7 days post-inoculation (dpi) (Fernández-Sainz et al., 2017). Furthermore, the Adt-O1Manisa vector in combination with Ad5-boIFNλ3, which drives the expression of bovine IFN-λ3, completely protects cattle against homologous challenge as early as 3 dpi (Diaz-San Segundo et al., 2016). On the contrary, in initial efficacy studies, the Ad5-O1/Campos/Brazil/58 (Ad5-O1C)-vectored vaccine has shown limited performance in target species such as swine (Caron et al., 2005). Consequently, several strategies have been implemented to improve Ad5-O1C vaccine performance. In this regard, the incorporation of the full-length coding sequence of the non-structural protein 2B (Ad5.O1C.2B) enhanced the FMDV-specific neutralizing antibody response in swine (Pena et al., 2008) and specific vaccine-induced T cell response in cattle (Moraes et al., 2011). On the other hand, the insertion of the RGD motif (Arg–Gly–Asp) into the adenovirus fiber protein (Adt.O1C.2B.RGD) did not significantly enhance vaccine efficacy in cattle (Medina et al., 2015). However, a similar adenovirus-vectored O1/Campos/Brazil/58 vaccine [Adt.O1C.2B.F(RGD)], but in combination with ENABL^®^ adjuvant, afforded complete protection of cattle against clinical FMD (Barrera et al., 2018). Given that naturally occurring empty capsids in infected cells are less abundant for most strains of serotype O than for serotype A (Rweyemamu et al., 1979), the lack of assembled empty capsid proteins might explain why adenovirus-vectored subunit vaccines for the O1/Campos/Brazil/58 strain are not as effective as the equivalents from serotype A or even other O subtypes.

Foot-and-mouth disease virus empty capsids are safe and as immunogenic as the intact viral particles (Rweyemamu et al., 1979). In fact, empty capsid particles are much more immunogenic than the individual capsid proteins or even partially assembled sub-viral particles (e.g., pentamers) or disrupted products (Rweyemamu et al., 1979; Doel and Chong, 1982). FMDV empty capsids comprise identical pentameric protein subunits held together by tenuous non-covalent interactions, which are unstable in mildly acidic pH conditions (Curry et al., 1995) or at elevated temperatures (Curry et al., 1997). In this respect, attempts to increase capsid acid or thermal stability by replacement of some residues by others that could strengthen inter-pentamer interactions have demonstrated to be an effective tool for the development of improved engineered vaccines against FMDV (Porta et al., 2013; Kotecha et al., 2015; Scott et al., 2017).

In the present study, we generated an optimized Ad5-vectored FMDV subunit vaccine against the O1/Campos/Brazil/58 strain by favoring the expression of FMDV capsid proteins and their self-association into virus-like particles. We analyzed several serological parameters after intramuscular inoculation with the optimized Ad5[P_VP2_]_OP_ candidate in a two-dose regimen formulated without adjuvants. Our results showed an enhanced vaccine performance, as demonstrated by the higher total and neutralizing antibody responses and an improved protection upon homologous challenge in a mouse model.

## Materials and Methods

### Cells and Viruses

Human embryonic kidney 293A (HEK 293A), Madin–Darby bovine kidney (MDBK), and baby hamster kidney 21 (BHK-21) cell lines were maintained in Dulbecco’s modified Eagle’s medium (DMEM) supplemented with 10% fetal bovine serum (FBS), 100 U/ml of penicillin G, and 100 μg/ml of streptomycin (Thermo Fisher Scientific). The recombinant Ad5 viruses used in this study included: Ad5[G], which expresses the enhanced green fluorescent protein (GFP) reporter gene (Romanutti et al., 2013); Ad5[P], which contains the P12A polyprotein capsid precursor and the 3C^pro^ coding regions (P12A3C) of the FMDV O1/Campos/Brazil/58 strain under the control of the cytomegalovirus enhancer-promoter (pCMV) (D’Antuono et al., 2010; Romanutti et al., 2013); and two novel Ad5 constructions denominated Ad5[P]_OP_ and Ad5[P_VP2_]_OP_, which are described below. The infectious FMDV strain O1/Campos/Brazil/58 used for challenge experiments was supplied by the Argentine FMD Reference Laboratory, Servicio Nacional de Sanidad y Calidad Agroalimentaria (SENASA). All experiments involving infectious virus were performed in the BSL4-OIE facilities at the Institute of Virology, CICVyA, INTA.

### Construction of Optimized Recombinant Adenoviruses

**FIGURE 1:**
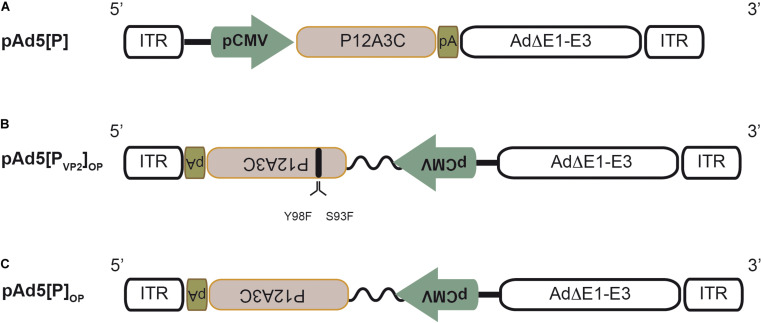
Schematic structure of the recombinant adenoviral genomes. **(A)** Recombinant pAd5[P] carrying the foot-and-mouth disease virus (FMDV) P12A3C cassette from O1/Campos strain (*brown box*) under the control of the cytomegalovirus enhancer-promoter (pCMV) in an E1/E3-deleted Ad5 backbone (Ad5ΔE1–E3). *pA*, SV40 poly(A) signal; *ITR*, inverted terminal repeats. **(B)** Recombinant pAd5[P_VP2_]_OP_. This Ad5 genome contains the expression unit at the E1 insertion site in a leftward orientation (3′→5′) in the ΔE1–E3 Ad5 backbone. The expression unit contains the FMDV P12A3C cassette under the control of the optimized pCMV (pCMV and human β-globin/IgG chimeric intron, *zigzag line*). Additionally, it harbors two non-synonymous substitutions in the VP2 coding sequence to express a S93F/Y98F VP2 mutant protein. **(C)** Recombinant pAd5[P]_OP_ is identical to pAd5[P_VP2_]_OP_, but expresses a native VP2 capsid protein.

Non-replicating (ΔE1/E3) optimized (OP) Ad5 vectors expressing O1/Campos/Brazil/58 (O1/Campos) FMDV capsid proteins were constructed through Gateway recombination following the manufacturer’s instructions (Gateway System, Thermo Fisher Scientific). Firstly, the pENTR[P]_OP_ shuttle plasmid was generated by inserting the pCMV, the chimeric β-globin/IgG intron, the P12A3C sequence, and the SV40 poly(A) signal from the pCI-P12A3C plasmid (Romanutti, 2012) between the *att*L1–*att*L2 recombination sites in pENTR^TM^4 (Thermo Fisher Scientific). Subsequently, amino acid substitutions were introduced into the FMDV VP2 full-length sequence using a QuikChange site-directed mutagenesis kit (Agilent Technologies) using pENTR[P]_OP_ as the template and primers containing the mutated sequences (S93Ff 5′-CAAAGGTGTCTACGGCTTCCTGACTG ACTCGTATGC-3′, S93Fr 5′-GCATACGAGTCAGTCAGGAA GCCGTAGACACCTTTG-3′, Y98Ff 5′-**C**CTGACTGACTCGTT TGCATATATGAGAAACGG-3′, and Y98Fr 5′-CCGTTTCT CATATATGCAAACGAGTCAGTCAGG-3′). The resulting plasmid, designated as pENTR[P_VP2_]_OP_, contains nucleotide changes (agc>ttc and tat>ttt) at positions corresponding to residues S93 and Y98 of the VP2 protein sequence. Finally, the pENTR[P_VP2_]_OP_ and pENTR[P]_OP_ shuttle vectors were recombined *in vitro* into the pAd/PL/V5-DEST^TM^ to obtain the pAd5[P_VP2_]_OP_ and pAd5[P]_OP_ genomes, respectively (Figures 1B,C). The correct insertion of the foreign sequences was confirmed by restriction digestion analysis and DNA sequencing. The final plasmids (pAd5[P_VP2_]_OP_ and pAd5[P]_OP_) were linearized with *Pac*I and transfected into HEK 293A cells using Lipofectamine 2000 (Thermo Fisher Scientific). The recombinant Ad5[P_VP2_]_OP_ and Ad5[P]_OP_ viruses were harvested 4–10 days post-transfection when approximately 80% cytopathic effect was observed. The Ad5 vectors were subsequently propagated and titrated into HEK 293A cells. Titration of Ad5 stocks was performed by the plaque-forming unit (PFU) method according to the manufacturer’s instructions (ViraPower^TM^ Adenoviral Expression System, Thermo Fisher). Vector titers were expressed as PFUs per milliliter. The average titers of the adenoviral stocks used in the experiments were as follows: Ad5[P], 7.5 × 10^9^ PFU/ml; Ad5[P]_OP_, 5.8 × 10^9^ PFU/ml; and Ad5[P_VP2_]_OP_, 5.2 × 10^9^ PFU/ml.

### *In vitro* FMDV Protein Expression Analysis

HEK 293A cells were infected with recombinant adenoviral particles at a multiplicity of infection (MOI) of 1. After 48 h incubation, the cells were washed with phosphate-buffered saline (PBS), lysed with 1× Laemmli buffer, and separated on 12% sodium dodecyl sulfate–polyacrylamide gel electrophoresis (SDS-PAGE). The fractionated proteins were transferred to nitrocellulose membranes and probed with the corresponding primary antibodies. Anti-FMDV O1/Campos polyclonal guinea pig serum (Seki et al., 2009) was used as the primary antibody to detect the production of recombinant capsid proteins. Anti-β-tubulin monoclonal antibody (mAb; AC-15, Sigma-Aldrich) was used as an internal control. Horseradish peroxidase (HRP)-conjugated anti-guinea pig or anti-mouse IgG were used as secondary antibodies (Cappel Laboratories Inc.). The blots were developed by using SupersignaL^®^ West Pico Chemiluminescent Substrate (Pierce), according to the manufacturer’s instructions. Protein band intensity was quantified by densitometry using ImageJ software (Abramoff et al., 2004).

### Purification of FMDV Subunits by Sucrose Gradients

HEK 293A and MDBK cells infected with Ad5 vectors at MOI 50 or 500, respectively, were lysed in NTE isotonic buffer (100 mM NaCl, 10 mM Tris–HCl, 1 mM EDTA, pH 7.4) containing 0.1% (*v*/*v*) Triton X-100 (Sigma-Aldrich). After 10 min incubation, the nuclei were removed by centrifugation and the supernatants were loaded onto 10–45% sucrose gradients in NTE and centrifuged at 22,000 rpm for 6 h in an SW28 rotor at 4°C. Density gradient fractions were collected and tested by enzyme-linked immunosorbent assay (ELISA) using non-cross-reactive anti-FMDV serotype O mAbs, as previously described (Seki et al., 2009). A stock of A/Argentina/2001 (A/Arg/01) inactivated FMD virus was also analyzed following the same protocol, but using an anti-A/Arg/01 polyclonal rabbit antiserum and specific mAbs against serotype A (Seki et al., 2009). Inactivated A/Arg/01 FMDV preparation was used as a marker of intact virions (146S), empty particles devoid of RNA (75S), and capsomer (12S) position in the gradient given the fact that the A/Arg/01 FMDV strain is able to generate a detectable amount of empty particles in cell cultures (Rowlands et al., 1975; Rweyemamu et al., 1979; Curry et al., 1995). For the detection of FMDV capsid proteins, eight fractions from the Ad5[P_VP2_]_OP_ gradient were selected and concentrated by trichloroacetic acid (TCA) precipitation (Link and Labaer, 2011). The resultant pellets were resuspended in 1× Laemmli buffer, applied to standard 12% SDS-PAGE gels, and then transferred onto nitrocellulose membranes. The membranes were probed with an anti-FMDV O1/Campos polyclonal guinea pig serum (Seki et al., 2009) followed by the corresponding HRP-conjugated secondary antibody.

For negative-staining transmission electron microscopy (TEM), fractions containing FMDV subunits were adsorbed to carbon-coated Parlodion films and mounted on 300-mesh/in. copper grids (EMS, Fort Washington, PA, United States) for 10 min, washed once with water, and stained for 1 min with 2% phosphotungstic acid, pH 7.0 (Sigma-Aldrich). The specimens were analyzed in a transmission electron microscope (Zeiss EM 109T) equipped with a CCD camera (Ultrascan ES1000W, Gatan, Pleasanton, CA, United States) at an acceleration voltage of 100 kV.

### Quantitative Analysis of FMDV Transcript Accumulation *in vitro* and *in vivo*

To examine the quantity of FMDV messenger RNAs (mRNAs) accumulated *in vitro*, HEK 293A cells were infected with Ad5-FMD vectors at a MOI of 5 in 12-well plates. Twenty-four hours post-infection (hpi), the cells were harvested in TRIzol (Thermo Fisher Scientific).

RNA was isolated following the manufacturer’s instructions and quantified using a NanoDrop spectrophotometer. Aliquots of 200 ng of purified total RNA were treated with RQ1 DNase, RNase-free (Promega) and then reverse-transcribed with SuperScript II reverse transcriptase (Thermo Fisher Scientific) using specific primers for the 3C^pro^ coding sequence (3Crt 5′-CTCGTGGTGTGGTTCG-3′) and human actin (Act_r, 5′-CGTCACCGGAGTCCATCACGA-3′) housekeeping gene. The primers for the amplification of the 3C^pro^ or actin 110-bp DNA fragment were designed by Primer Express^®^ software v3.0.1 (Applied Biosystems). Quantitative reverse transcription polymerase chain reactions (RT-qPCRs) were run in triplicate using 1/10th of the diluted complementary DNA (cDNA), 300 nmol of each primer (3Cf 5′-AAGATGGTCATGGGCA ACAC-3′ and 3Cr 5′-CGAGGTAAGCAGTGCCAAAC-3′ or Act_f 5′-GAGACCTTCAACACCCCAGCC-3′ and Act_r), and 10 μl of 2× FastStart Universal SYBR Green Master (Rox; Roche) in a final volume of 20 μl. PCR cycle conditions were set as follows: pre-incubation for 10 min at 95°C followed by 40 cycles, each including 15 s at 95°C and 60 s at 60°C. Dissociation curves were generated at the end of the run to verify the specificity of the reaction product. Relative quantification was performed by Applied Biosystems 7500 Real-Time PCR software v2.0.6 (Thermo Fisher Scientific) based on “cycle threshold” (Ct) values. Average 3C^pro^ Ct values were normalized to the average Ct values for actin, and the relative level of 3C^pro^ (*R*) was estimated as ΔΔCt-based fold change using the 2^–ΔΔ*Ct*^ equation.

For transcript accumulation *in vivo* analysis, BALB/cJ mice were inoculated by the intramuscular (i.m.) route with 5 × 10^8^ PFU of either the prior Ad5[P] or the optimized Ad5[P_VP2_]_OP_ viruses (three animals per group). For transgene transcript accumulation analysis by RT-qPCR, draining popliteal lymph nodes (dPLNs) were isolated at 2 and 8 dpi, and cell suspensions were obtained in TRIzol (Thermo Fisher Scientific). cDNA synthesis was performed as described above, but using specific primers for 3C^pro^ (3Crt) and glyceraldehyde-3-phosphate dehydrogenase (GAPDHqr, 5′-CAGAAGGTGCGGAGATGATGA-3′) included as the housekeeping gene. RT-qPCR reactions were also run in triplicate using 1/10th of the diluted cDNA, 300 nmol of each primer (3Cf and 3Cr or GAPDHqf 5′-TGCTGGTGCCGAGTATGTTG-3′ and GAPDHqr). Average 3C^pro^ Ct values were normalized to the average Ct values for GAPDH, and the relative level of 3C^pro^ (*R*) was estimated as ΔΔCt-based fold change using the 2^–ΔΔ*Ct*^ equation.

### Experimental Animals and Immunization Protocols

In the present study, a murine model was employed as a predictor of the immunogenicity and protection induced by Ad5-based FMD vaccines (Bidart et al., 2020; Gnazzo et al., 2020). The experiments carried out in mice reported in this manuscript have been performed following internationally recognized guidelines with the approval of the Institutional Committee for Care and Use of Experimental Animals, CICUAE (approval reference CICUAE CEVAN 01.1/2020, 02.1/2020, and INTA-CICVyA 10/2020).

Groups of 16 mice (4- to 6-week-old male BALB/cJ mice) were injected i.m. with 50 μl of the Ad5[P_VP2_]_OP_ vaccine candidate (5 × 10^8^ PFU per animal). For comparison purposes, three additional groups of mice were inoculated with Ad5[P]_OP_ (*n* = 16), Ad5[P] (*n* = 8), or a commercial oil-adjuvanted inactivated O1/Campos strain vaccine (IV; *n* = 12). Animals inoculated with a recombinant Ad5 expressing GFP (Ad5[G]), considered as an unrelated antigen, were also included (*n* = 8). According to the 3R’s (replacement, reduction, and refinement), and due to ethical limitations, the number of animals used in each experimental group was reduced when possible, as in the case of groups inoculated with Ad5[P], Ad5[G], and IV. These groups were included only as control groups since they were already evaluated in previous works (D’Antuono et al., 2010; Romanutti et al., 2013). Table 1 summarizes the treatments given to the different groups of mice. Individual serum samples were collected from all mice at −2, 21, 35, and 45 dpi. Animal experiments were conducted in duplicate.

**TABLE 1:** Mice immunization schedule.

Immunogens^*a*^					
Prime (day 0)	Boost (day 28)	Adjuvant	Dose (PFU)	Inoculation route	Challenge^*b*^ (day 49)
Ad5[P]_OP_	Ad5[P]_OP_	–	5 × 10^8^	i.m.	Yes
Ad5[P_VP2_]_OP_	Ad5[P_VP2_]_OP_	–	5 × 10^8^	i.m.	Yes
Ad5[P]	Ad5[P]	–	5 × 10^8^	i.m.	Yes
Ad5[G]	Ad5[G]	–	5 × 10^8^	i.m.	Yes
PBS	IV	+	1 μg	i.p.	Yes

*IV, oil-adjuvanted inactivated commercial O1/Campos strain vaccine (Aftogen; BiogenesisBago, Argentina); P, P12A3C sequence from FMDV O1/Campos; G, green fluorescent protein; PFU, plaque-forming units. ^a^Detail of the immunogens used in each experimental group. ^b^10^4.5^ TCID_50_ of FMD infective virus.*

### Determination of FMDV-Specific Antibodies Titers

Anti-FMDV antibody titers were estimated by ELISA, following a modified protocol from Seki et al. (2009). Briefly, ELISA microplates (MaxiSorp^TM^, Nunc) were coated with a rabbit antiserum against the FMDV O1/Campos strain in carbonate–bicarbonate buffer (pH 9.6) and incubated overnight (ON) at 4°C. The inactivated O1/Campos 146S particles were then added to the wells and incubated for 1 h at 37°C. Subsequent steps were performed using a blocking buffer (PBS containing 3% horse serum and 0.05% Tween-20). Murine sera to be tested were serially diluted in blocking buffer and subsequently added to the coated ELISA plate and incubated for 1 h at 37°C. Peroxidase-labeled anti-mouse IgG, IgG1, or IgG2a antibodies were used to develop the reactions. Antibody (Ab) titers were expressed as the log_10_ of the reciprocal of serum dilutions giving at least twice the absorbance at 405 nm estimated in sera from animals inoculated with Ad5[G], which represents the negative control condition. Isotyping of the Ab response was carried out in sera at 45 dpi.

To examine the antigen avidity index, the plates were coated ON at 4°C with purified O1/Campos FMDV 146S particles in 50 mM carbonate/bicarbonate buffer (pH 9.6). After five washes with PBS-T (PBS with 0.05% Tween-20), 50 μl of a single dilution of serum samples (1/50) was added and incubated for 1 h at 37°C. The plates were washed twice with PBS-T and subsequently incubated with PBS–4 M urea for 15 min at room temperature, followed by two PBS-T washing steps. FMDV-specific antibodies were detected with the HRP-labeled anti-mouse conjugate incubated for 1 h at 37°C. The colorimetric reaction was revealed with the chromogen/substrate mixture ABTS/H_2_O_2_ at room temperature. The reaction was stopped after 30 min by the addition of 2% sodium fluoride and the absorbance was read at 405 nm. The optical density (OD) values of the samples were corrected by subtracting the mean blank OD values (cOD). The avidity index (AI) was calculated as the percentage of residual activity of each serum relative to the cOD of the untreated (not washed with urea) sample: AI% = (cOD sample with urea/cOD sample without urea) × 100 (Lavoria et al., 2012; Romanutti et al., 2013; Bidart et al., 2020).

### Virus Neutralization Test

Sera were examined for anti-FMDV O1/Campos neutralizing antibodies (nAbs) as described before (Quattrocchi et al., 2014). Briefly, serial dilutions of complement inactivated sera were incubated for 1 h at 37°C with 100 fifty percent tissue culture infective dose (TCID_50_) of the infective FMDV O1/Campos. Then, virus–serum mixtures were seeded on BHK-21 monolayers. After 40 min at 37°C, fresh DMEM 2% fetal calf serum (FCS) was added to the monolayers, which were incubated at 37°C under 5% CO_2_. Cytopathic effects were observed after 48 h. Titers are expressed as log_10_ of the reciprocal of the serum dilution that neutralizes 50% of 100 TCID_50_ of the infective FMDV O1/Campos using the fixed virus–variable serum method.

### Virus Challenge

Mice were challenged by intraperitoneal (i.p.) inoculation of 10^4.5^ TCID_50_/ml of infective FMDV O1/Campos strain per mouse. The groups of mice described in Table 1 were challenged at 49 dpi in BSL4-OIE facilities and analyzed for the presence of viremia 24 h post-challenge. Protection against FMDV was assessed as previously described, with minor modifications (Carrillo et al., 1998; Quattrocchi et al., 2011). Twenty-four hours after challenge (50 dpi), the animals were anesthetized and bled by the retro-orbital route. Heparinized blood was spread undiluted on BHK-21 cell monolayers; after virus adsorption, the monolayers were washed with sterile PBS. Fresh DMEM with 2% FCS was added and the cells were kept for 48 h at 37°C in a 5% CO_2_ incubator. An animal was considered protected if the cell monolayer did not present a cytopathic effect after a blind passage. Protection percentages were calculated as *P*% = (number of protected mice/number of challenged mice) × 100.

### Bone Marrow Dendritic Cells Isolation

Bone marrow was obtained from the femurs and tibias of 8-week-old C57BL/6J males by flushing RPMI 1640 medium through the bone interior. Then, red blood cells were lysed and the cells were cultured at 37°C and 5% CO_2_ in six-well plates (1.5 × 10^6^ cells per well) in 2 ml of RPMI 1640 containing 10% FBS, 50 μg/ml gentamycin (Sigma), and 20 ng/ml of recombinant GM-CSF (GenScript). The media was renovated on days 3 and 5, and on day 8 the cells were harvested and the percentage of bone marrow dendritic cells (BMDCs) was determined by the co-expression of CD11c and major histocompatibility complex (MHC) class II surface markers.

### BMDCs Activation

Bone marrow dendritic cells (5 × 10^5^ cells/well) were stimulated with 500 ng/ml lipopolysaccharide (LPS) as a positive control condition or infected with Ad5[P] or Ad5[P_VP2_]_OP_ at different MOIs (50 and 250) for 1 h at 37°C and 5% CO_2_ in RPMI 1640. Then, the supernatant was removed and RPMI 1640 containing 10% FBS was added and the cells were incubated at 37°C and 5% CO_2_. After 24 h, the cells were washed and resuspended in 100 μl of ice-cold PBS–azide plus anti-FcγR mAb (CD16/32; clone 93) and incubated for 30 min on ice. Then, APC anti-mouse CD11c (clone M1/70), FITC anti-mouse MHC-II (clone M5/114.15.2), PE anti-mouse CD80 (clone 16-10AI), and PerCP anti-mouse CD86 (clone PO3) mAbs were added in the recommended concentrations and incubated for additional 40 min on ice. Next, the cells were washed, fixed with 1% *p*-formaldehyde in PBS, and analyzed by flow cytometry. FlowMax cytometer PASIII (Partec, Münster, Germany) and Flowjo software (FlowJo, Ashland, OR, United States) were used for the analysis. All mAbs and their isotype controls were from Biolegend.

### Sequence Analysis and Prediction of FMDV VP2 Structure

All sequences were retrieved by BLAST using FMDV O1/Campos VP2 (Uniprot accession no. Q8UZC1) as the query in the NCBI non-redundant database. Multiple sequence alignments were done by t_coffee (Di Tommaso et al., 2011) on a set of 116 non-redundant VP2 sequences and on a subset of 22 representative VP2 sequences. Amino acid conservation scores were retrieved from The ConSurf Server^1^ (Berezin et al., 2004; Ashkenazy et al., 2016). Both alignments exhibited comparable conservation scores at the analyzed positions. Data are available upon request. A three-dimensional (3D) model of the O1/Campos VP2 protein was created with MODELLER (Marti-Renom et al., 2000; Eswar et al., 2006) based on the full-length FMDV O1 crystal structure [Protein Data Bank (PDB) code 1QQP.3] (Fry et al., 1993). Validation of the model was carried out by ProSA (Wiederstein and Sippl, 2007) and Verify3d (Eisenberg et al., 1997). Also, the stereochemical quality of the model was assessed by the Ramachandran plot using PROCHECK (Laskowski et al., 1993). Superimposition (Maiti et al., 2004) with 1QQP.3 gave a global root mean square deviation (RMSD) of 0.32 Å across 216 equivalent positions. The interfacing region at the inter-pentamer contact of FMDV O1/Campos VP2 was modeled using the PDB coordinates 1BBT and 5DDJ as templates. All the evaluations supported the correctness of the models.

### Statistical Analysis

Statistical analysis was performed using the GraphPad Prism 6 software (CA, United States). Comparison between mean values of the two groups (i.e., mean Ab titers) was assessed by unpaired non-parametric Mann–Whitney test. Comparison of the RNA levels in infected cells and dPLNs as well as the activation marker levels among the different Ad5-infected BMDCs was performed using two-way analysis of variance (ANOVA). Differences were considered significant for *p* < 0.05.

## Results

### Rational Design of Optimized Recombinant Ad5 Viruses

Previously, we have described an adenovirus vector (Ad5[P]) that contains the FMDV O1/Campos P12A polyprotein and 3C^pro^ coding regions placed under the control of the pCMV into the E1 locus in the rightward orientation (5′→3′) relative to the Ad5 genome (D’Antuono et al., 2010; Romanutti et al., 2013). Ad5[P] (Figure 1A) was able to express and properly process the FMDV capsid proteins, but it was unable to induce protective immunity in a murine experimental model (D’Antuono et al., 2010; Romanutti et al., 2013). Therefore, to improve the Ad5-vectored O1/Campos subunit vaccine performance, we explored the impact of increasing the expression levels of FMDV capsid proteins as well as improving their ability to self-associate into VLPs on vaccine immunogenicity and efficacy. Accordingly, we engineered an optimized novel Ad5 vector that contains modifications in the FMDV autonomous expression unit.

**FIGURE 2:**
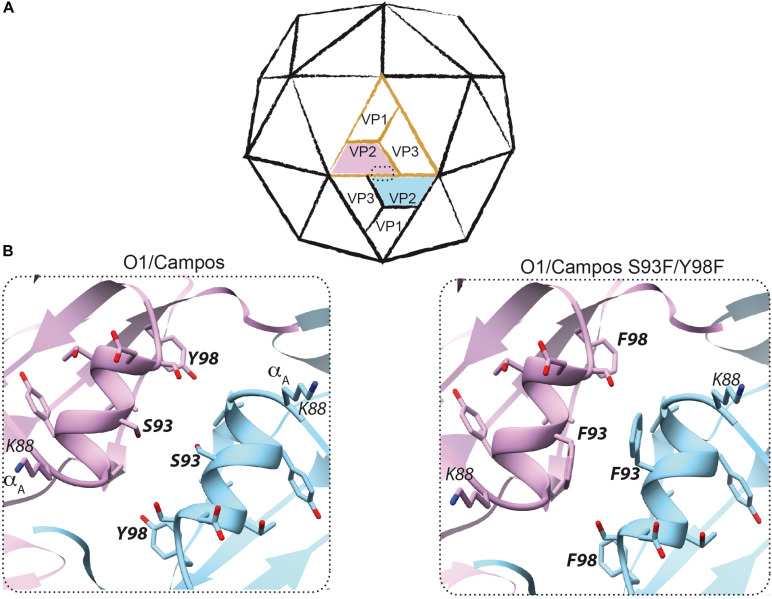
Rational design to produce empty capsids of foot-and-mouth disease virus (FMDV) O1/Campos. **(A)** Scheme of the icosahedral FMDV capsid. A protomer consisting of VP1, VP2, VP3, and the internal polypeptide VP4 (not visible) is shown in *orange*. The interface between two pentamers at the two-fold symmetry axis, where two neighboring VP2–VP2 interact, is enclosed *within a dashed rectangle*. **(B)** The VP2 structure of FMDV O1/Campos was modeled as described in the section “Materials and Methods”. This model led to the prediction and potential localization of residues S93 and Y98, located on the α-helix A (amino acids K88–Y98) at the two-fold symmetry axis.

Firstly, to enhance the expression of the O1/Campos capsid proteins, we placed the P12A3C transgene under the transcriptional control of an optimized pCMV, which comprises the pCMV and the human chimeric β-globin/IgG intron regulatory element, which is well known to produce strong intron-mediated enhancement-related effects on mRNA expression (Buchman and Berg, 1988; Wu et al., 2008; Choi et al., 2014). Additionally, in order to avoid aberrant splicing between the transgene and the adenoviral genes, the FMDV expression unit was embedded into the E1 locus in a leftward orientation (3′→5′) (Rubinchik et al., 2001; Nakai et al., 2007; Suzuki et al., 2015). Secondly, to favor the self-assembly of the FMDV O1/Campos capsid proteins into VLPs, we introduced substitutions in amino acids located in the N-terminal of the VP2 capsid protein, whose side chains are engaged in inter-pentamer interactions. In particular, the replacements S93F and Y98F were described as essential to increase the pH and thermal stability of the less stable FMD O1/Manisa and SAT2 virus particles and recombinant empty capsids (Kotecha et al., 2015; Rincon et al., 2015; Ganji et al., 2018). We surmised that introducing these mutations in both residues would favor O1/Campos empty capsid formation *in situ*. Therefore, we defined the presence and disposition of these relevant residues involved in VP2–VP2 contacts at the capsid inter-pentamer interfaces. To that end, we generated a molecular model of the VP2 monomer employing the 3D structure of FMDV O1BFS (99% sequence identity) as a template (PDB 1BBT). Based on the location in this model, as well as on the conservation score of the individual positions across seven representative FMDV O subtypes in multiple sequence alignments (data not shown), strictly conserved amino acids S93 and Y98 were corroborated as candidates for mutagenesis (Figure 2). By this rationale, we generated the Ad5[P_VP2_]_OP_ vaccine candidate that contains the leftward-directed FMDV expression unit under the control of the optimized pCMV. Additionally, it carries two non-synonymous substitutions in the VP2 coding sequence to express a S93F/Y98F VP2 mutant protein (Figure 1B).

Complementary, to evaluate specifically the contribution of the introduced double mutation upon the *in vitro* VLP assembly as well as its influence on the induction of adaptive immune response in mice, we also generated the Ad5[P]_OP_ vector to be used as a control. Regarding expression unit structure and orientation, this vector is identical to Ad5[P_VP2_]_OP_, but expresses a wild-type VP2 capsid protein (Figure 1C).

### Expression of FMDV O1/Campos Capsid Proteins in Mammalian Cells

**FIGURE 3:**
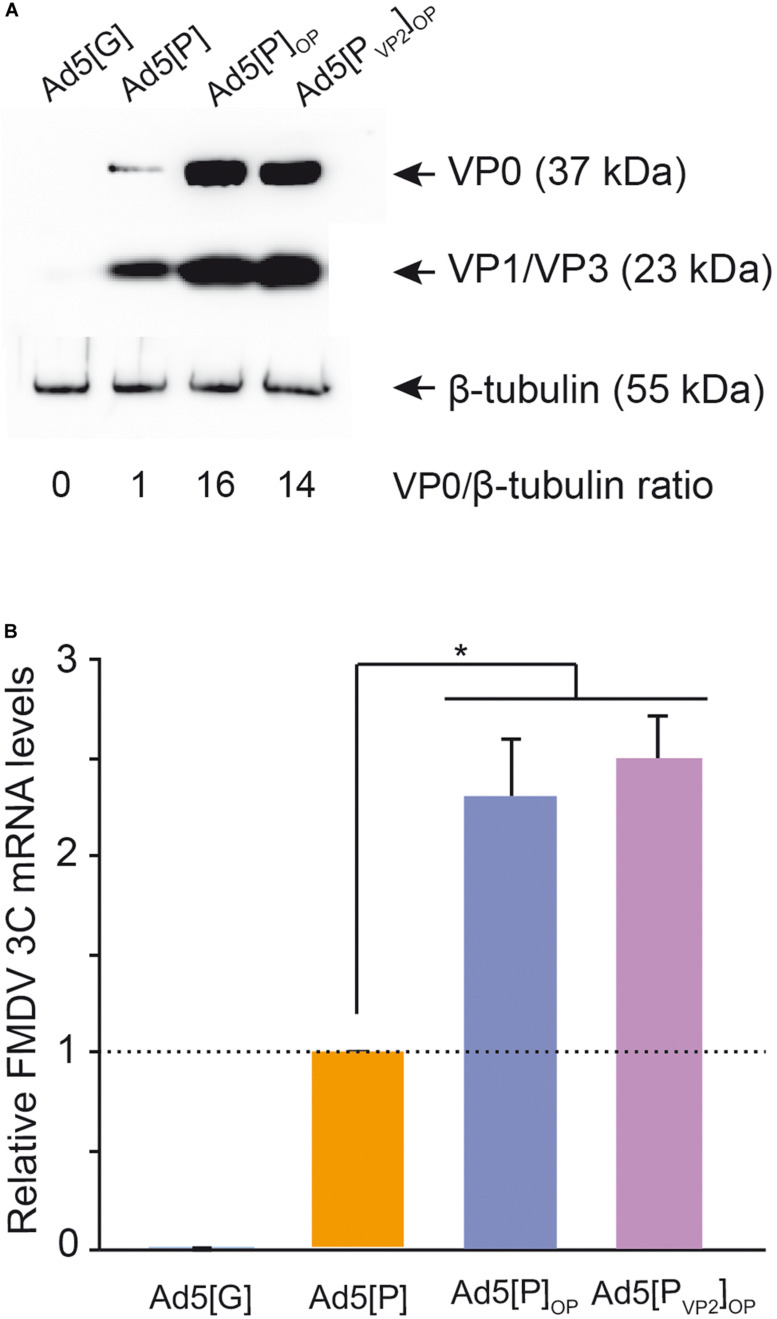
Analysis of foot-and-mouth disease virus (FMDV) capsid protein expression. Lysates of human embryonic kidney (HEK) 293A cells infected with Ad5[P], Ad5[P]_OP_, or Ad5[P_VP2_]_OP_ were analyzed by SDS-PAGE and Western blot using FMDV anti-O1/Campos polyclonal serum **(A)**. An Ad5 encoding an irrelevant antigen (green fluorescent protein), Ad5[G], was also included. To estimate FMDV structural protein accumulation, the ratios of VP0 to β-tubulin bands were calculated and normalized to the Ad5[P] VP0/β-tubulin ratio, set at 1.0. Mean values from two independent experiments are presented. Standard deviations ranged from 0% to 2% (not shown). VP0, VP1/VP3, and β-tubulin are indicated with an *arrow*. **(B)** Analysis of 3C mRNA expression in HEK 293A cells infected with Ad5[G], Ad5[P], Ad5[P]_OP_, or Ad5[P_VP2_]_OP_ by quantitative real-time PCR. HEK 293A cells were infected with the corresponding Ad5 vector at multiplicity of infection (MOI) 5. Twenty-four hours post-infection, total RNA was isolated and used to amplify a fragment of the FMDV 3C^pro^ open reading frame (ORF) and, as a control, a fragment of human Actin ORF by RT-qPCR. For each experimental group, the FMDV 3C^pro^ transcript level relative to the Ad5[P] group was calculated by using the 2^− ΔΔ*Ct*^ equation. Data correspond to the average and standard deviation from two independent experiments. *p < 0.01 (ANOVA).

In the first place, we evaluated the impact of the optimization approach in FMDV capsid protein expression. To that end, HEK 293A cells were infected with Ad5[P_VP2_]_OP_ or Ad5[P]_OP_, and FMDV capsid protein accumulation was analyzed by Western blot using a polyclonal Ab against O1/Campos FMDV (Figure 3A). As expected, neither VP0, VP3, nor VP1 was detected upon infection with Ad5[G] (lane 1). A discrete band corresponding to the mature capsid proteins VP0, VP1, and VP3 was identified in lysates from Ad5[P]-, Ad5[P]_OP_-, and Ad5[P_VP2_]_OP_-infected cells (lane 2, 3, and 4, respectively), indicating that these vectors are able to deliver the expression of FMDV polyprotein, which is properly processed. Interestingly, cells infected with either the Ad5[P_VP2_]_OP_ or Ad5[P]_OP_ vector also yielded a quantitative stronger signal than those cells infected with Ad5[P], indicative of an increased capsid protein accumulation. Quantification by ELISA (data not shown) and densitometry (samples infected at a MOI of 1) indicated an approximately 14-fold increase in VP0 accumulation in cells infected with Ad5[P_VP2_]_OP_ or Ad5[P]_OP_ vectors as compared to Ad5[P] (Figure 3A). Moreover, no remarkable differences on the FMDV protein expression levels were observed between the cells infected with Ad5[P]_OP_ or Ad5[P_VP2_]_OP_ (lanes 3 and 4, respectively). Consistently, HEK 293A cells infected with the Ad5[P]_OP_ or Ad5[P_VP2_]_OP_ vectors accumulated significantly higher levels of 3C^pro^ mRNA (two-fold increase) when compared to those infected with the Ad5[P] vector (Figure 3B).

In brief, these results showed that the insertion of the optimized leftward-directed FMDV expression unit boosts FMDV capsid protein expression through enhanced FMDV transcript levels delivered by the Ad5 vector. This phenomenon does not correlate with the introduction of S93F and Y98F substitutions within the VP2 capsid protein.

### Characterization of Capsid-Like Particles Assembled in Ad5[P_VP2_]_OP_-Infected Cells

Subsequently, the effect of the targeted amino acid mutations on the FMDV VP2 capsid protein in the self-assembly of empty capsids was investigated. In preliminary experiments, we found that ultracentrifugation in sucrose gradients of lysates derived from non-permissive MDBK cells infected with Ad5[P_VP2_]_OP_ showed a sedimentation pattern consistent with the generation of 75S FMDV subunits (Supplementary Figure S1).

**FIGURE 4:**
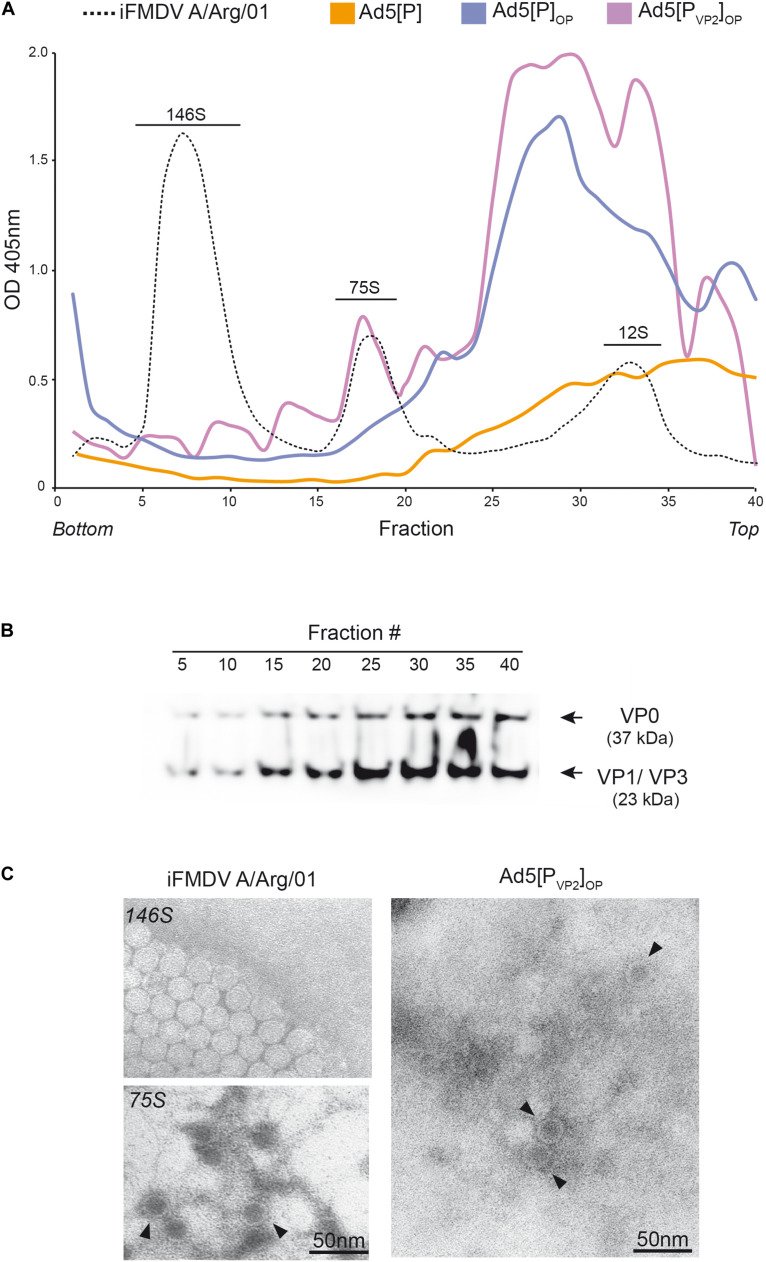
Assembly of recombinant capsid proteins into subviral particles. **(A)** Lysates of HEK 293A cells infected with Ad5[P], Ad5[P]_OP_, or Ad5[P_VP2_]_OP_ were loaded onto 10–45% sucrose gradients. Inactivated native A/Argentina/2001 foot-and-mouth disease virus (iFMDV A/Arg/01) was also included as a marker (*black dotted line*). Fractions were collected after centrifugation at high speed and analyzed by ELISA. The positions of the FMDV virions (146S), empty capsids (75S), and capsomers (12S) are indicated. **(B)** Eight fractions (5, 10, 15, 20, 25, 30, 35, and 40) were analyzed by Western blot with specific anti-FMDV O1/Campos polyclonal antibody. VP0 and VP1/VP3 are indicated with an *arrow*. **(C)** Negative-staining TEM of *in situ* generated FMDV empty capsid particles (*right*) and iFMDV 146S and 75S particles (*left*). *Scale bar*, 50 nm. Virus-like particles are indicated (*filled inverted triangle*).

To confirm whether this finding was specific for the VP2-introduced mutations, lysates from HEK 293A cells infected with Ad5[P_VP2_]_OP_, Ad5[P], or Ad5[P]_OP_ were analyzed by sucrose gradient ultracentrifugation and the resulting fractions were examined using serotype-specific antigen ELISAs (Seki et al., 2009). Considering that FMDV O1/Campos does not produce easily detectable native empty particles in cell cultures, inactivated FMDV A/Argentina/2001 (A/Arg/01) was analyzed in parallel as a marker for 146S, 75S, and 12S particles. In lysates from cells infected with Ad5[P], Ad5[P]_OP_, or Ad5[P_VP2_]_OP_, FMDV capsid proteins predominantly associated in macromolecular complexes that sedimented at a lower rate than did the empty capsids (fractions 23–36) (Figure 4A). However, in Ad5[P_VP2_]_OP_-infected cell lysates, a fraction of the self-assembled subunits was detected at the same rate as the native empty FMDV strain A/Arg/01 capsids (75S). The absence of the 75S fraction in the lysates from cells infected with Ad5[P]_OP_ is indicative that amino acid substitutions S93F and Y98F (introduced into the VP2 sequence) favor FMDV empty capsid particles assembly *in situ*. Distribution analysis of the FMDV capsid proteins throughout the sucrose gradient from HEK 293A cells infected with Ad5[P_VP2_]_OP_ by Western blot revealed specific bands corresponding to VP0 (37 kDa) and VP1/VP3 (23 kDa) (Figure 4B). We roughly quantified the amount of VLPs produced in cells infected with the Ad5[P_VP2_]_OP_ vector by estimating the area under the curve. According to this method, VLPs represented approximately 12 ± 5% of the total antigenic material produced in infected cells. These results were confirmed by indirect ELISA using a linear standard curve derived from a reference 146S O1/Campos FMDV (data not shown).

Finally, fractions were obtained from the middle of the gradients (fractions 15–20) and were analyzed by negative-staining TEM. VLPs of approximately 30 nm in diameter could be observed in the fractions derived from HEK 293A cells infected with the Ad5[P_VP2_]_OP_ candidate (Figure 4C, right). These structures were indistinguishable from the native empty viral particles generated by A/Arg/01 (Figure 4C, left). Thus, the Ad5[P_VP2_]_OP_ vaccine candidate favors the production of uniform round O1/Campos VLPs with a diameter of about 30 nm, which is similar to the average diameter of the authentic FMDV particles.

### *In vivo* Expression of FMDV Transgene

**FIGURE 5:**
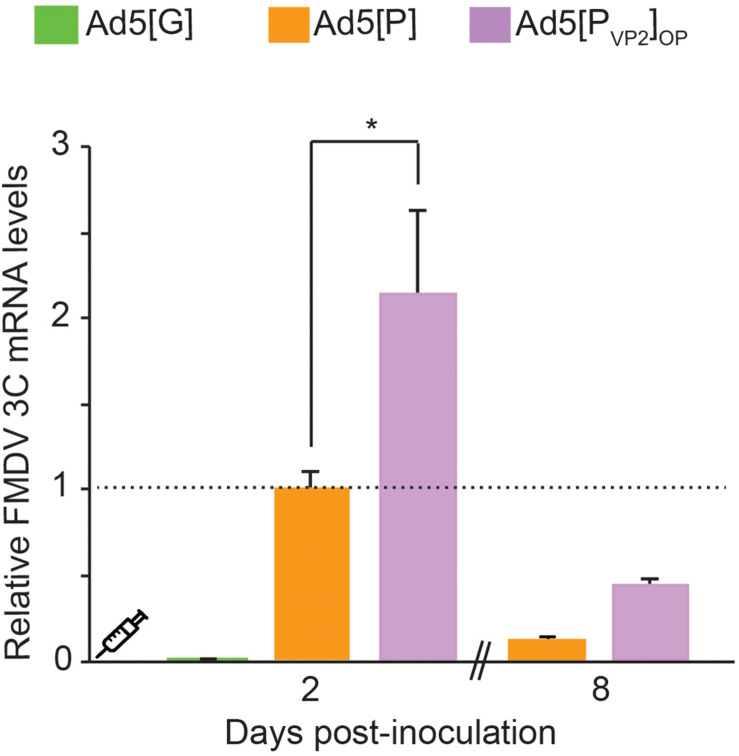
Comparative detection of foot-and-mouth disease virus (FMDV) transcripts in draining popliteal lymph nodes (dPLNs) of inoculated mice. Comparison of transcript accumulation levels in dPLNs after inoculation of BALB/cJ mice. Animals (*n* = 3) were inoculated i.m. with 5 × 10^8^ PFU of Ad5[G], Ad5[P], or Ad5[P_VP2_]_OP_. Total RNA was extracted from dPLNs at 2 and 8 dpi, respectively, and a fragment of the FMDV 3C^pro^ open reading frame (ORF) and the GAPDH ORF were amplified by RT-qPCR. For each experimental group, the FMDV 3C^pro^ transcript level relative to the Ad5[P] group was calculated by using the 2^− ΔΔ*Ct*^ equation. Data correspond to the average and standard deviation from two independent experiments. *p < 0.01 (ANOVA).

Our *in vitro* results indicated that the Ad5[P_VP2_]_OP_ vaccine candidate is more efficient in supporting FMDV capsid protein expression than the initial Ad5[P] vector. Thereafter, we tested whether the use of the leftward-directed FMDV expression unit containing the optimized pCMV would enhance FMDV mRNA accumulation *in vivo*. To that end, mice were inoculated with Ad5[G], Ad5[P], or Ad5[P_VP2_]_OP_ and FMDV 3C^pro^ transcript accumulation was assessed at 2 and 8 dpi in mouse dPLNs by RT-qPCR (Figure 5). At 2 dpi, animals inoculated with the Ad5[P_VP2_]_OP_ candidate accumulated approximately twofold increased levels of 3C^pro^ transcripts compared with those inoculated with the previous Ad5[P] (*p* < 0.01). Furthermore, despite a decline, 3C^pro^ transcripts were still detected in mice inoculated with the Ad5[P_VP2_]_OP_ candidate at 8 dpi. These data indicate that the Ad5[P_VP2_]_OP_ vaccine candidate also drives the expression of higher levels of FMDV mRNA than the Ad5[P] vector *in vivo*.

### Antibody Response After Immunization With Recombinant Ad5 Vectors

**FIGURE 6:**
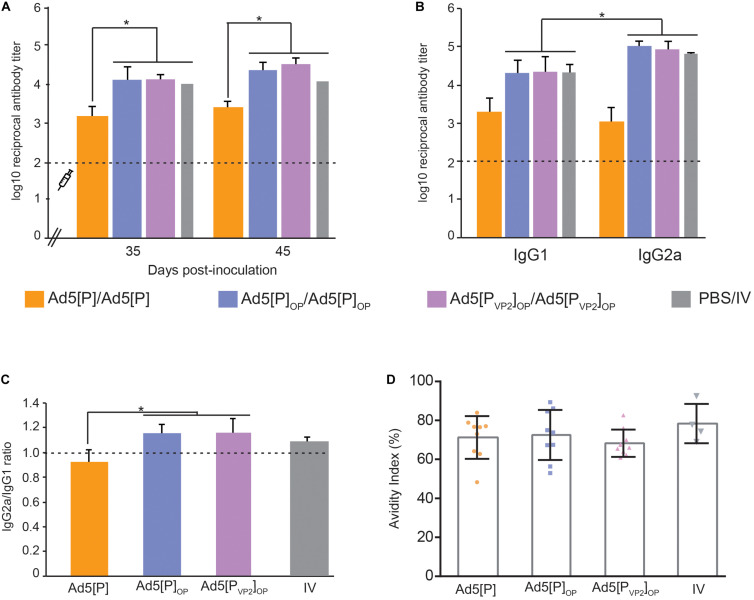
Immune responses in mice after the administration of Ad5[P]_OP_ and Ad5[P_VP2_]_OP_ particles. **(A)** Kinetics of foot-and-mouth disease (FMD) antigen-specific antibodies in BALB/cJ mice. The *syringe* indicates the booster inoculation (at 28 dpi). *Asterisks* indicate statistical significance in antibody titers (*p* < 0.05). FMD virus (FMDV)-specific antibody titers were determined by enzyme-linked immunosorbent assay (ELISA) in sera from animals bled at 35 and 45 days post-inoculation. **(B)** Isotype antibody profile induced by different Ad5 candidates. ELISA titers of anti-FMDV-specific IgG1 or IgG2a determined at 45 dpi are shown. *Asterisk* indicates the statistically significant difference between the IgG1 and IgG2a levels (*p* < 0.05). **(C)** IgG2a/IgG1 ratio measured in each experimental group. The consistency of the IgG ratios was assessed through the determination of the significant differences in the titers of both isotypes in each group (shown at the *top of the bars*). **(D)** Avidity of the antibodies induced by different immunogens. Serum samples from inoculated mice at 45 dpi were used to estimate the avidity index as a percentage. The average index corresponded to the optical density (OD) 405 nm of the urea-treated samples divided by the OD 405 nm of those PBS-treated. *Dots* correspond to values from individual animals of the indicated group; the mean value is indicated (*solid line*).

Immunogenicity experiments comparing the prior Ad5[P] vector and the newly optimized Ad5[P_VP2_]_OP_ vaccine candidate were assessed in mice following the inoculation schedule shown in Table 1. In order to understand the nature of the immune response afforded by Ad5[P_VP2_]_OP_, we also analyzed the immunogenicity of the assembly deficient Ad5[P]_OP_ vector. Anti-FMDV endpoint ELISA was performed with serum from individual animals bled at −2, 21, 35, and 45 dpi. As was previously reported (D’Antuono et al., 2010; Romanutti et al., 2013), after the first dose (21 dpi), significant differences in specific Ab titers could not be established between the different experimental groups of mice (data not shown). The Ab titers significantly increased in all groups after a booster immunization (35 dpi), with no further increase at 45 dpi (Figure 6A). Animals primed and boosted with the optimized vector Ad5[P_VP2_]_OP_ or Ad5[P]_OP_ developed specific FMDV Ab titers that were significantly higher than the ones elicited by the animals inoculated with Ad5[P] and similar to those by the animals primed with the oil-adjuvanted inactivated O1/Campos FMDV vaccine (*p* < 0.05). As expected, the administration of a vector encoding an unrelated antigen, Ad5[G], did not elicit measurable anti-FMDV antibodies (not shown).

The Ab isotype analysis at 45 dpi revealed that the sera from animals vaccinated with both optimized vectors induced higher FMDV-specific IgG1 and IgG2a titers than Ad5[P], but interestingly, Ad5[P_VP2_]_OP_ and Ad5[P]_OP_ evoked IgG2a as the predominant isotype (*p* < 0.05; Figures 6B,C), similar to the animals vaccinated with the oil-adjuvanted inactivated vaccine. Therefore, Ad5[P_VP2_]_OP_ and Ad5[P]_OP_ seemed likely to induce a Th1-dependent humoral immune response and showed the highest IgG2a-to-IgG1 ratio of all the experimental groups.

To extend this analysis, we compared the avidity of specific FMDV antibodies in each experimental group. Specific antibodies elicited at 45 dpi by the inoculation of Ad5[P_VP2_]_OP_, Ad5[P]_OP_, or Ad5[P] had similar high avidity indexes (>50%), which did not differ significantly from those promoted by the inactivated conventional FMDV vaccine (Figure 6D).

As a whole, these data indicate that the expression levels of FMDV antigens driven by the Ad5[P_VP2_]_OP_ and Ad5[P]_OP_ vectors were sufficient to elicit a strong immune response comparable to the oil-adjuvanted inactivated O1/Campos FMDV commercial vaccine.

### Protection From Challenge

**FIGURE 7:**
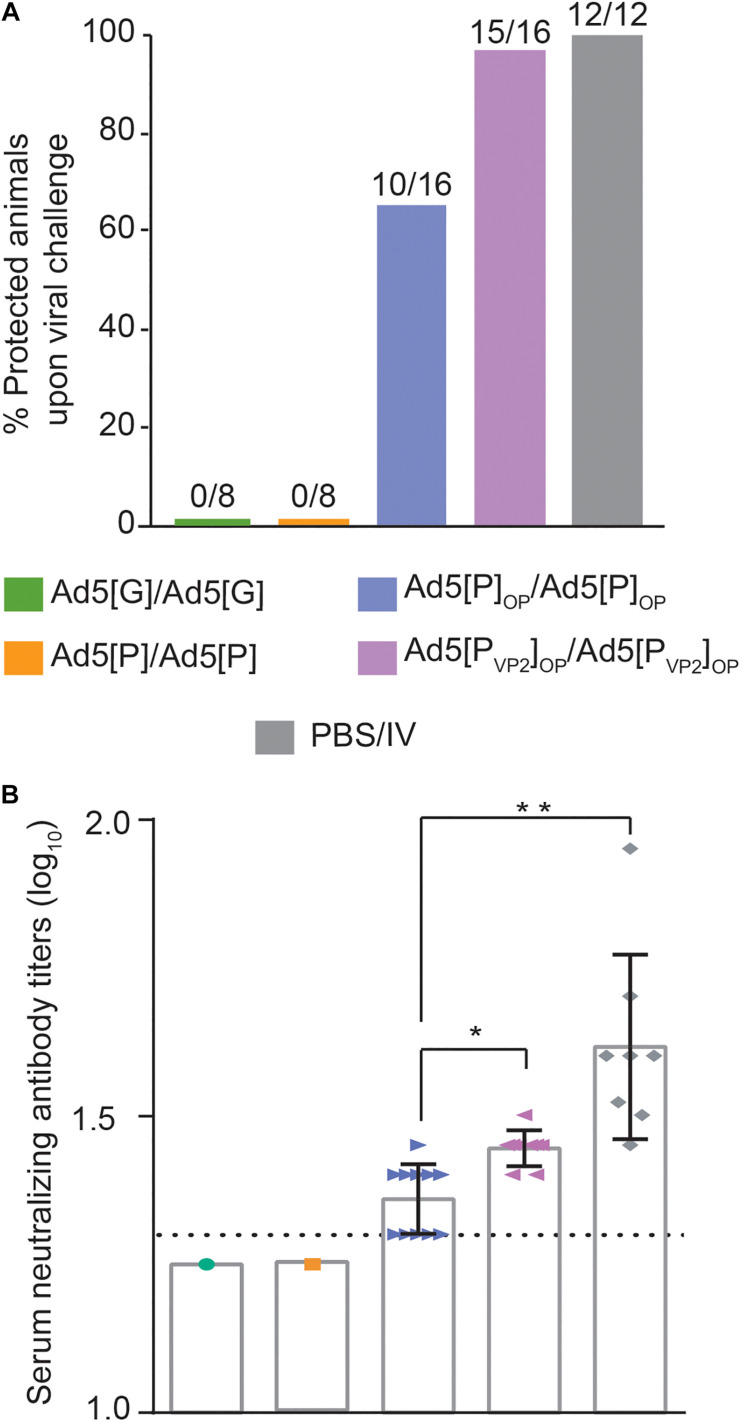
Protection against challenge with infectious foot-and-mouth disease virus (FMDV) O1/Campos. **(A)** Protection from viral challenge elicited by the different Ad5-FMD vectors. Groups of mice were inoculated with Ad5[P], Ad5[P]_OP_, Ad5[P_VP2_]_OP_, Ad5[G], or a commercial FMD vaccine and challenged with infective FMDV at 49 dpi. Animals were considered protected if viremia was absent after a blind passage. Protection percentages were calculated as 100 × (number of vaccinated animals without viremia/number of vaccinated animals). **(B)** Virus neutralization test (VNT) at day 45 post-inoculation with 5 × 10^8^ PFU, expressed as log_10_ of the reciprocal of the serum dilution that neutralizes 50% of 100 TCID_50_ of infective FMDV O1/Campos (fixed virus–variable serum method). *p < 0.05, **p < 0.01 with respect to the Ad5[P] group. A serum pool was made every two animals of a group in order to perform the serum neutralization test.

According to the immunization scheme shown in Table 1, animals were challenged at 49 dpi with the infective O1/Campos virus strain and were analyzed for the presence of viremia 24 h post-challenge. As shown in Figure 7A, protection was found in 62.5% (10 out of 16) of the Ad5[P]_OP_ group and 94% (15 out of 16) of the Ad5[P_VP2_]_OP_ group. No protection (0%) was found in the groups inoculated with Ad5[P] or Ad5[G], and 100% protection was observed in animals inoculated with one dose of the inactivated commercial FMDV vaccine (12 out of 12).

Animals vaccinated with either the Ad5[P_VP2_]_OP_ or Ad5[P]_OP_ vector developed specific FMDV-neutralizing Ab responses (Figure 7B). As expected, the highest nAb titers were found in mice inoculated with the inactivated commercial FMDV vaccine (1.60 ± 0.15). However, the mean of the nAb titer of the group inoculated with Ad5[P]_OP_ remained near the test detection limit (1.36 ± 0.02), while the mean titer of the group inoculated with the Ad5[P_VP2_]_OP_ vaccine candidate (1.45 ± 0.03) was significantly higher than that of Ad5[P]_OP_ (*p* = 0.0175) and was consistent with the afforded protection (Figure 7A). For animals of the non-protected groups (Ad5[P] and Ad5[G]), the nAb titers were found to be below the assay detection limit (<1.3).

### *In vitro* Stimulation of BMDCs With Ad5[P] and Ad5[P_VP2_]_OP_

**FIGURE 8:**
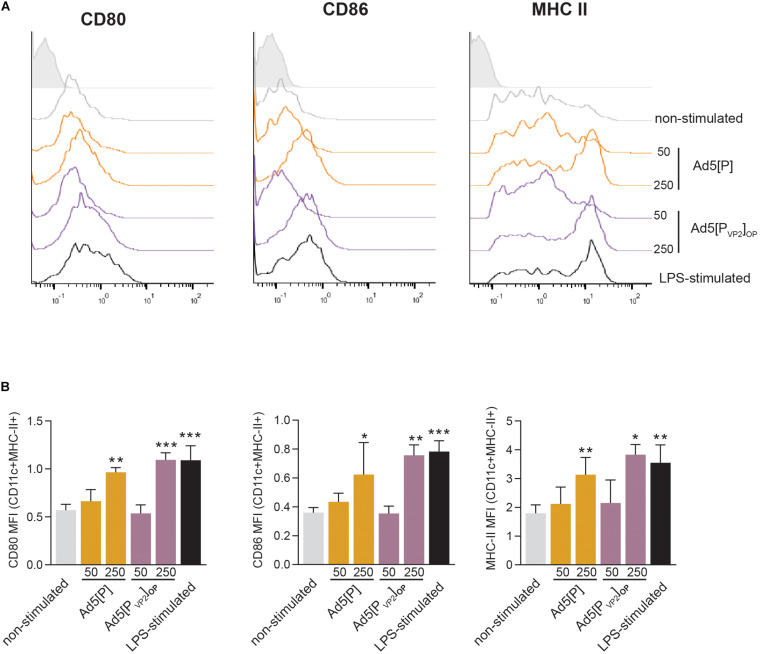
Bone marrow dendritic cell (BMDC) activation induced by Ad5[P] or Ad5[P_VP2_]_OP_ infection. BMDCs were infected with Ad5[P] or Ad5[P_VP2_]_OP_ at multiplicity of infection (MOI) of 50 and 250, as described in the section “Materials and Methods.” Then, the cells were stained with specific monoclonal antibodies (mAbs) for phenotypic and activation markers and analyzed by flow cytometry. **(A)** Representative histograms depict the fluorescence intensities of CD80, CD86, and major histocompatibility complex (MHC) class II molecules on MHCII^+^CD11c^+^ gated population. **(B)** *Bars* represent the geometric mean of fluorescence intensity (MFI) ± SD. *Isotype*, cells were stained with control isotype mAbs; *LPS-stimulated*, cells were treated with 500 ng/ml lipopolysaccharide (LPS). *p < 0.05, **p < 0.01, ***p < 0.001 compared to non-stimulated cells (ANOVA).

Dendritic cells are potent antigen-presenting cells that initiate and modulate the host immune response. Our *in vivo* data showed that Ad5[P_VP2_]_OP_ is highly immunogenic and is the most efficient in conferring protection after homologous challenge. Since Ad5[P] and Ad5[P_VP2_]_OP_ are antigenically equivalent and only differ in their ability to deliver FMDV subunits, we sought to determine the effect of these gene transfer vectors on the activation phenotype of mouse BMDCs. To that end, BMDCs were infected with Ad5[P] or Ad5[P_VP2_]_OP_ at different MOIs (50 or 250) or mock-infected. Twenty-four hours later, the BMDC phenotype was assessed by using flow cytometry analysis for CD80, CD86, and MHC-II surface expression. As expected, treatment of mouse BMDCs with LPS, included as a maturation control, resulted in a significant upregulation of MHC-II, CD80, and CD86 expression (Figure 8). Infection of mouse BMDCs with Ad5[P] or Ad5[P_VP2_]_OP_ at MOI 50 did not induce the upregulation of any of the studied surface markers. In contrast, infection with Ad5[P] or Ad5[P_VP2_]_OP_ at MOI 250 led to a twofold increase of the MHC-II, CD80, and CD86 expression levels as compared to non-infected BMDCs. Overall, these results suggest that BMDC activation depended on a threshold dose since no effect on maturation was apparent at a MOI of 50, and the maturation rate at a MOI of 250 was similar to that induced by LPS. Notably, both Ad5[P] and Ad5[P_VP2_]_OP_ stimulated a strong activation of mouse BMDCs.

## Discussion

Although recombinant adenovirus vectors have demonstrated promising capabilities as vaccine candidates for several FMDV strains (Grubman et al., 2009), it has been more difficult to achieve the same results with some strains of serotype O, specifically for the epidemiologically relevant O1/Campos/Brazil/58 strain (Caron et al., 2005; D’Antuono et al., 2010; Moraes et al., 2011; Romanutti et al., 2013; Medina et al., 2015).

It is known that the performance of FMDV vaccines is dependent on the amount of intact viral particles (146S) (Terry et al., 1982) or natural empty capsids (75S) (Rweyemamu et al., 1989) in the vaccine’s preparation. In this study, we explored the hypothesis that enhanced expression of the FMDV capsid proteins and their self-association into VLPs would improve the immunogenicity and efficacy of the Ad5-FMD O1/Campos vaccine. Thereby, a novel recombinant Ad5-vectored vaccine (Ad5[P_VP2_]_OP_) was engineered by modifying the sense of transcription of the foreign expression unit and by expressing the FMDV proteins from an optimized pCMV. The hypothesis was further co-implemented with the introduction of specific amino acid replacements (S93F and Y98F) within the N-terminal of VP2, reported to stabilize the generated empty capsids (Kotecha et al., 2015).

The *in vitro* data reported in this work support the working hypothesis and revealed a substantial increase (∼14-fold) in FMDV capsid protein accumulation in cells transduced with the optimized Ad5[P_VP2_]_OP_ (and also Ad5[P]_OP_) vector compared with those cells transduced by our group’s first-generation vector, Ad5[P] (Figure 3A). Moreover, the Ad5[P_VP2_]_OP_ vaccine candidate was able to deliver an increased FMDV transcript accumulation (Figure 3B). Therefore, these results showed that the insertion of the heterologous transcription unit in the leftward orientation relative to the Ad5 genome, and the expression of FMDV sequences from an optimized pCMV, led to an increase in mRNA and capsid protein accumulation levels in transduced cells. The data was very relevant since Pena et al. (2008) reported a 1.5- to 1.7-fold increase when using an identical CMV enhancer/promoter and chimeric intron combination to drive the expression of FMDV A24 Cruzeiro capsid proteins, indicating that the positioning of the optimized P12A3C expression unit in an opposite transcriptional orientation relative to the Ad5 genome is a suitable additional strategy for optimizing FMDV gene expression *in vitro* (Rubinchik et al., 2001; Nakai et al., 2007; Suzuki et al., 2015).

As predicted, the Ad5[P_VP2_]_OP_ vaccine candidate was able to drive the assembly of FMDV O1/Campos VLPs in infected permissive and non-permissive cells, while Ad5[P] and Ad5[P]_OP_ were not. Sucrose gradients of Ad5[P_VP2_]_OP_-infected cells showed a FMDV protein fraction sedimenting at the same rate as the native empty particles (75S) of the A/Arg/01 strain produced in culture (Figure 4A and Supplementary Figure S1). Indeed, examination by negative-staining TEM revealed that the O1/Campos VLPs closely resemble the native FMD empty capsid particles in size and overall particle morphology (Figure 4C). Thus, the introduced double-phenylalanine substitution at positions S93 and Y98 increased the yields of assembly of FMDV O1/Campos empty capsids. The phenotype exhibited by this mutated VP2 confirmed previously reported results indicating that a 10-residue region spanning α-helix A (amino acids 88–98 for the O serotype) of VP2 protein is relevant for the infectivity and stability of FMDV (Ellard et al., 1999; Porta et al., 2013; Kotecha et al., 2015; Ganji et al., 2018). Furthermore, these studies have shown that residues within this region are responsible for non-covalent interactions between adjacent pentamers at the icosahedral two-fold axis, where neighboring VP2–VP2 proteins interact. As shown in the O1/Campos VP2 model, these residues are likely committed to local inter-pentameric subunit associations (Figure 2).

The serological data reinforce the initial hypothesis since the amount of FMDV transgene expressed by Ad5[P_VP2_]_OP_ (and Ad5[P]_OP_) is sufficient to elicit a strong immune response in mice, comparable to the oil-adjuvanted inactivated O1/Campos FMDV vaccine. It is of note that the humoral and protective responses against FMDV detected in our murine model have a good established correlation with cattle (Habiela et al., 2014; Bidart et al., 2020; Gnazzo et al., 2020). We demonstrated that the Ad5[P_VP2_]_OP_ vaccine candidate elicited an enhanced antibody response as compared to our first-generation vector, Ad5[P] (Figures 6A, 7B). Notably, 94% of the animals inoculated with Ad5[P_VP2_]_OP_ were completely protected from a severe FMD virus challenge (Figure 7A), which suggests that, at a similar dose of the tried Ad5-vectored vaccines, Ad5[P_VP2_]_OP_ provides additional expression of the FMDV proteins and/or ability in antigen presentation to immune cells sufficient to protect the vaccinated animals. It is known that a higher antigenic mass of the O1/Campos strain is required to achieve equivalent potency in inactivated vaccines as compared to other serotype antigens (Doel et al., 1994; Doel, 2003; Pena et al., 2008). It appears that the efficacy of the Ad5-vectored O1/Campos vaccine is also dependent on the amount of transgene expressed in transduced cells. In this regard, significant differences in transgene expression were detected in the dPLN populations isolated from mice inoculated with Ad5[P_VP2_]_OP_ or Ad5[P] (Figure 5). While the levels of RNA do not necessarily correlate with the protein expression levels determined *in vitro* (Figure 3), these differences in FMDV RNA levels could explain the improved efficacy and the higher potency elicited by the Ad5[P_VP2_]_OP_ candidate *in vivo* as compared to the Ad5[P] vector.

The levels of neutralizing antibodies were markedly higher in mice inoculated with Ad5[P_VP2_]_OP_ than in those inoculated with Ad5[P] (Figure 7B). Although serum-neutralizing antibodies have been considered the most relevant parameter that correlates with protection against FMDV (Doel, 2003; Paton et al., 2005; World Organisation for Animal Health, 2012), the contribution of non-neutralizing antibodies in the protection against infection and disease has also been described (McCullough et al., 1992; Pay and Hingley, 1992; Dunn et al., 1998). In this sense, higher levels of isotype IgG1 and IgG2a specific antibodies were quantified in the groups inoculated with the optimized vectors than in those mice inoculated with Ad5[P] (Figures 6B,C). These increased IgG1 and IgG2a specific antibodies may have played a relevant role in FMDV opsonization and its subsequent clearance by phagocytic cells of the immune system (McCullough et al., 1988). Further studies to explore the contribution of other mechanisms involved in the protection induced by Ad5[P_VP2_]_OP_ in the mouse model, as the induction of a detectable T cell response, are needed.

Dendritic cells (DCs) play an important role in both the initiation of innate immunity and the development of adaptive immunity (Hochrein and O’Keeffe, 2008). Previous reports have demonstrated that recombinant Ad5 induces the maturation of dendritic cells by upregulating the expression of major histocompatibility complex class I and II antigens, co-stimulatory molecules (CD40, CD80, and CD86), and the adhesion molecule CD54 (ICAM-1) (Morelli et al., 2000). The preliminary shown in this work suggested that BMDCs infected with Ad5[P] or Ad5[P_VP2_]_OP_ upregulated the expression of MHC class II and co-stimulatory molecules (CD80 and CD86) to a similar extent (Figure 8). This data adds evidence to previous reports that demonstrated that an unequal relationship exists between the rate of transgene expression and the extent of DC maturation (Morelli et al., 2000; Miller et al., 2003; Philpott et al., 2004). FMDV structural and non-structural proteins have been shown to antagonize the host innate response in mammalian cells by preventing the translational (Guzylack-Piriou et al., 2006) or transcriptional (de Los Santos et al., 2006) regulation of host proteins. Although Ad5 vectors expressing FMDV proteins seem to induce BMDCs to adopt an activated phenotype, it remains unclear whether other biological functions of these cells, which include antigen uptake, migration to regional lymph nodes, and antigen-specific T-lymphocyte activation, are affected.

When the optimized vectors were compared in mouse challenge experiments, a remarkable finding was the ability of the Ad5[P_VP2_]_OP_ candidate to confer higher protection than Ad5[P]_OP_ (94% and 60%, respectively). Evaluation of specific parameters of the humoral immune response such as total specific antibody titers, isotype balance, and specific antibody avidity indicated no significant difference in these attributes (Figure 6). In this scenario, differences in other responses such as innate and cellular immunity cannot be discarded. For example, an increased antibody-dependent phagocytosis (Lannes et al., 2012; Quattrocchi et al., 2011) and T CD8-mediated cytotoxicity (Guzman et al., 2010; Patch et al., 2013) may exert a synergistic effect, explaining the higher protection afforded by Ad5[P_VP2_]_OP_. Furthermore, our results may indicate that elements in addition to transgene expression levels are likely to have the potential to significantly influence the enhanced resistance to challenge in Ad5[P_VP2_]_OP_-vaccinated animals. It is then tempting to suggest that the superior ability of Ad5[P_VP2_]_OP_ in the assembly of FMDV VLPs shown *in vitro* may have accounted for its enhanced efficacy *in vivo*.

One of the foremost factors reported to influence the potency of vaccine preparations is the structural integrity of the whole (146S) FMD viral particles, mainly due to the capacity of this antigen to elicit antibodies against exposed epitopes that can drive to viral clearance in the case of a natural infection (Cartwright et al., 1982; Doel and Chong, 1982; Rao et al., 1994). In particular, neutralizing antibody production is associated mainly with 146S particles (Bahnemann, 1975; Pay and Hingley, 1992), whereas disrupted viral particles (12S pentamers) are less immunogenic and elicit a diminished neutralizing response (Doel and Chong, 1982; Rao et al., 1994). It has recently been demonstrated that FMDV-specific production of IFN-γ is strongly affected by the integrity of the viral capsids (Bucafusco et al., 2015). Additionally, FMDV-specific IFN-γ production in vaccinated cattle has been associated with the activation of antigen-specific CD4^+^ T cells (Oh et al., 2012), which are responsible for supporting antibody-neutralizing responses (Carr et al., 2013). In this regard, a comparative analysis of the antigenic reactivity with a panel of 13 mAbs specific for FMDV serotype O (Seki et al., 2009) revealed that the FMDV subunits launched by Ad5[P_VP2_]_OP_ exhibit an improved reactivity with neutralizing and non-neutralizing mAbs as compared to those antigens generated by the assembly deficient Ad5[P]_OP_ (Supplementary Figure S2). Moreover, we demonstrated that the Ad5[P_VP2_]_OP_ vaccine candidate elicited moderate but significantly higher titers of nAb than did Ad5[P]_OP_ (Figure 7B). Therefore, it is tempting to speculate that FMDV subunits generated by the Ad5[P_VP2_]_OP_ vaccine candidate form an oligomeric protein scaffold capable of presenting immunogenic epitopes and proteins in a more efficient manner, similar to the native FMD virion.

Traditional inactivated FMDV vaccines have played and still play a preponderant role in preventing and controlling the disease worldwide (Caridi et al., 2017; Khalifa et al., 2017). However, the drawbacks associated with its production, the duration of immunity, the necessity of a strict cold chain to preserve particle stability, among others, have led to continuous attempts to develop a large variety of alternative FMD vaccines. In this respect, we have generated the optimized Ad5[P_VP2_]_OP_ candidate which launches the expression of high levels of FMDV O1/Campos subunits that partially self-associate into VLPs. The results herein demonstrated the remarkable antigenicity and efficacy of this platform in a very well-supported murine model. Notwithstanding, the potency of the Ad5[P_VP2_]_OP_ vaccine candidate should be further studied in target species, such as pigs or cattle, whenever possible.

## Data Availability Statement

The raw data supporting the conclusions of this article will be made available by the authors, without undue reservation.

## Ethics Statement

The animal study was reviewed and approved by 1. Institutional Committee for Care and Use of Experimental Animals (CICUAE) Affiliation: Instituto de Virología, Centro de Investigaciones en Ciencias Veterinarias, Instituto Nacional de Tecnología Agropecuaria, (INTA-CICVyA), N. Repetto y De Los Reseros s/n, Hurlingham (1686), Consejo Nacional de Investigaciones Científicas y Técnicas (CONICET), Buenos Aires, Argentina. 2. Institutional Committee for Care and Use of Experimental Animals (CIEMAE) Affiliation: Centro de Virología Animal, Consejo Nacional de Investigaciones Científicas y Técnicas (CEVAN-CONICET), Saladillo 2468, C1440FFX, Buenos Aires, Argentina.

## Author Contributions

AD and NM developed the idea and design of the study. MZ, JB, and CP performed the experiments. MZ and AD analyzed the data and interpreted the results. AD and NM wrote the original draft. MZ, JB, CP, PZ, MT, and NM wrote and critically revised the manuscript. All authors read and approved the final manuscript.

## Conflict of Interest

The authors declare that the research was conducted in the absence of any commercial or financial relationships that could be construed as a potential conflict of interest.

## Footnotes

1 https://consurf.tau.ac.il/

## Supplementary Material

Supplementary Figure 1 Assembly of recombinant structural proteins into subviral particles. Lysates of MDBK cells infected with Ad5[P_VP2_]_OP_ were loaded onto 10–45% sucrose gradients. Fractions were collected and analyzed by ELISA. Inactivated native A/Argentina/2001 FMDV (iFMDV A/Arg/01) was included as a marker (black dotted line). The positions of the FMDV virions (146S), empty capsids (75S) and capsomers (12S) are indicated.

Supplementary Figure 2 Antigenic reactivity of FMDV subunits launched by Ad5[P]_OP_ and Ad5[P_VP2_]_OP_. Lysates of HEK 293A cells infected with Ad5[P]_OP_ or Ad5[P_VP2_]_OP_ were analyzed by using specific mAbs for O1/Campos FMDV by ELISA. The reactivities of FMDV subunits against several mAbs are shown as the absorbance values (OD 405 nm) detected for each specific mAb. The antigenic reactivity for inactivated O1/Campos virus (iFMDV O1/Campos) was included as reference sample.
