# Supplementary figures and images for: Optimized Adenoviral Vector That Enhances the Assembly of FMDV O1 Virus-Like Particles in situ Increases Its Potential as Vaccine for Serotype O Viruses
Source: Front Microbiol. 2020 Nov 4;11:591019. doi: 10.3389/fmicb.2020.591019 (PMC7672010; doi:10.3389/fmicb.2020.591019)

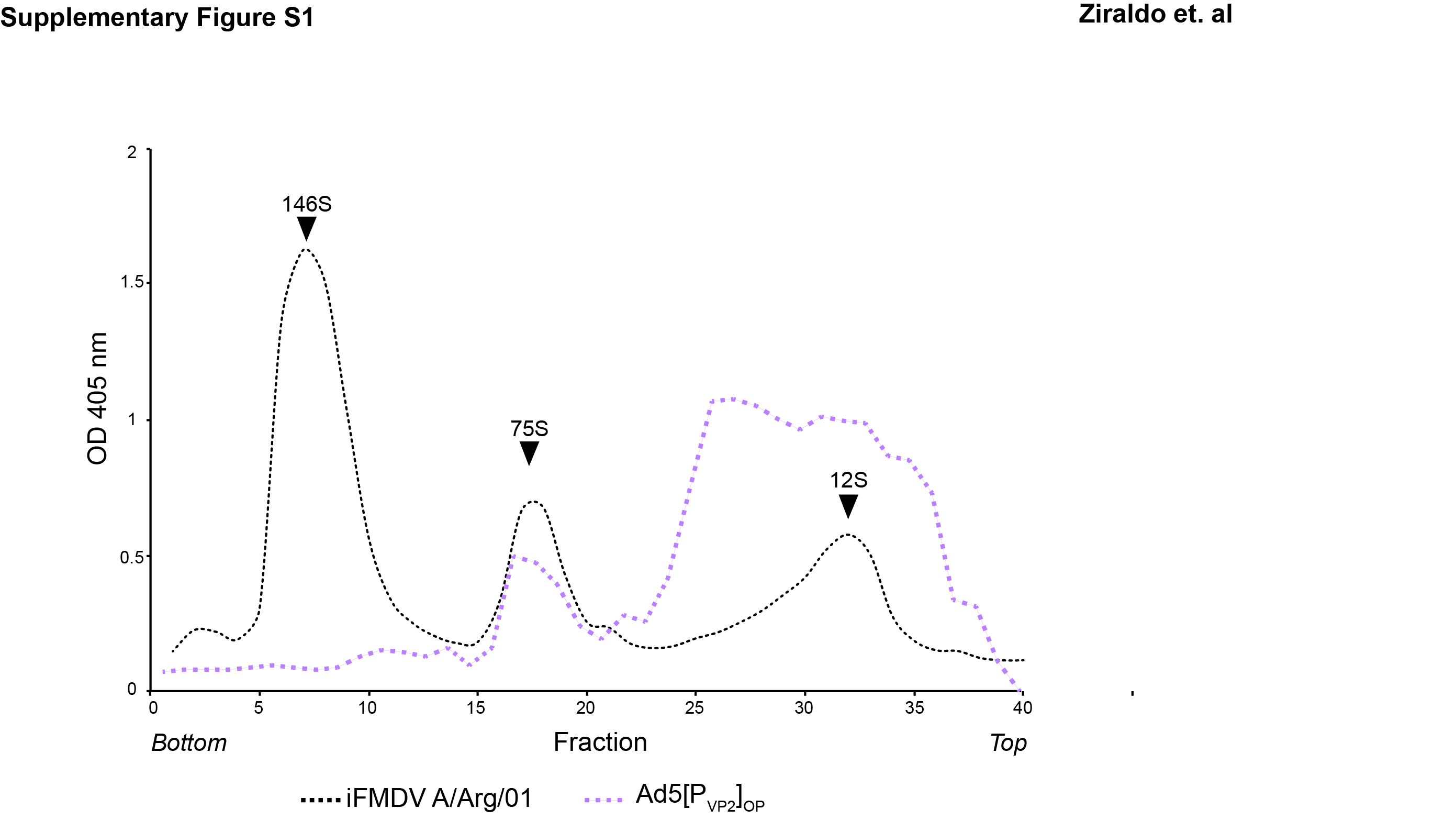

Supplement: Supplementary Figure 1 — Assembly of recombinant structural proteins into subviral particles. Lysates of MDBK cells infected with Ad5[PVP2]OP were loaded onto 10–45% sucrose gradients. Fractions were collected and analyzed by ELISA. Inactivated native A/Argentina/2001 FMDV (iFMDV A/Arg/01) was included as a marker (black dotted line). The positions of the FMDV virions (146S), empty capsids (75S) and capsomers (12S) are indicated. [file Image_1.JPEG]

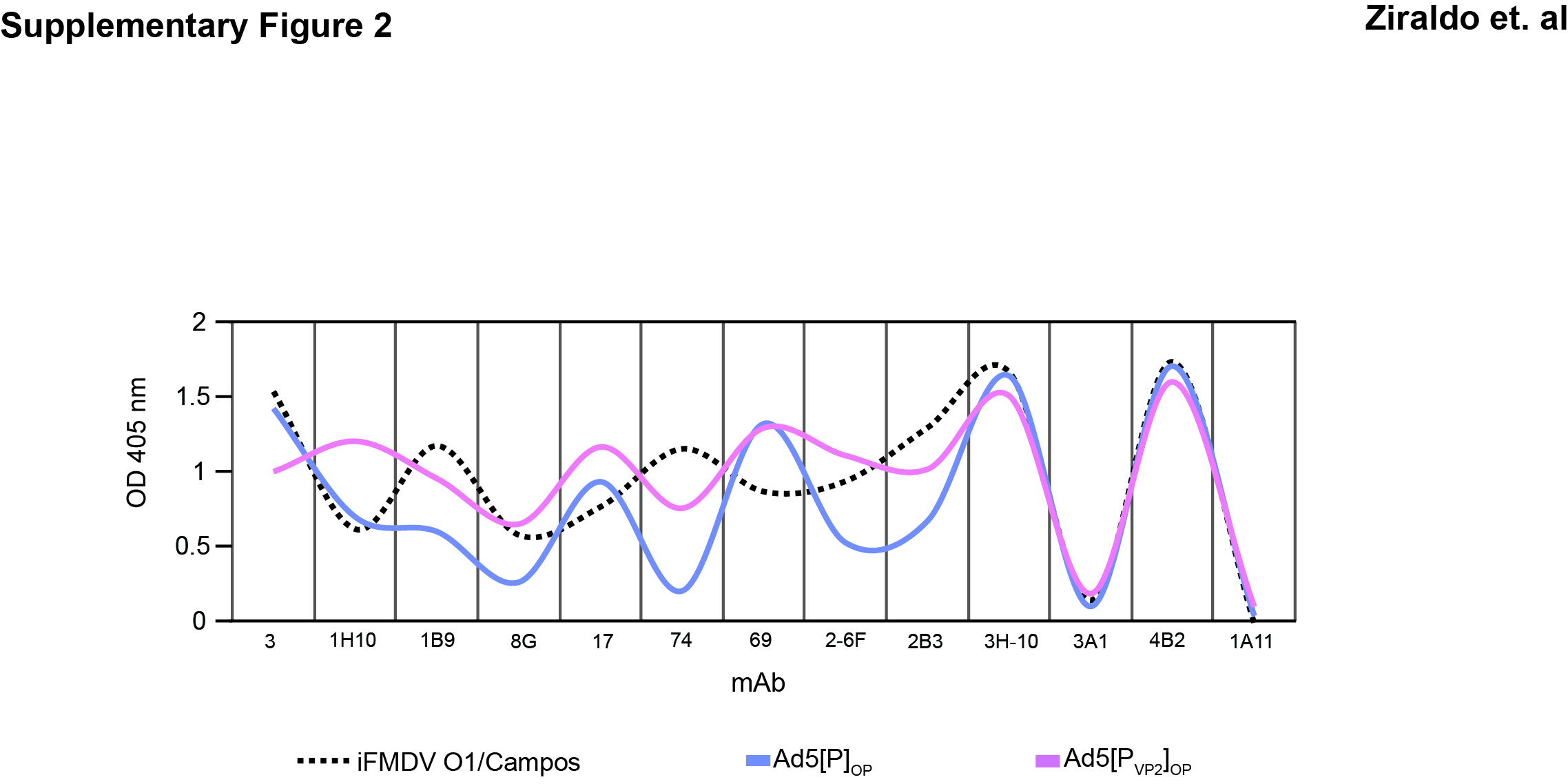

Supplement: Supplementary Figure 2 — Antigenic reactivity of FMDV subunits launched by Ad5[P]OP and Ad5[PVP2]OP. Lysates of HEK 293A cells infected with Ad5[P]OP or Ad5[PVP2]OP were analyzed by using specific mAbs for O1/Campos FMDV by ELISA. The reactivities of FMDV subunits against several mAbs are shown as the absorbance values (OD 405 nm) detected for each specific mAb. The antigenic reactivity for inactivated O1/Campos virus (iFMDV O1/Campos) was included as reference sample. [file Image_2.JPEG]
